# Supplementary material for: Are carbon emissions trading and green financial instruments synergistic? -Comprehensive quantitative research based on content analysis
Source: PLoS One. 2024 Mar 7;19(3):e0298601. doi: 10.1371/journal.pone.0298601 (PMC10919601; doi:10.1371/journal.pone.0298601)
Supplement: S1 Data — (ZIP) [file pone.0298601.s001.zip › Data/地级市Z_F工作报告.zip!/╜¡╦╒/╤∩╓▌╩╨/└┤╫╘╣1⁄2╓┌║┼í╛┬φ┐╦╩2╛▌═°í┐_2006.docx]

各位代表：

现在，我代表扬州市人民政府，向大会作政府工作报告，请予审议，并请市政协各位委员和其他列席人员提出意见。

2005年和“十五”时期工作回顾

过去的一年，我们在中共扬州市委的领导下，依靠和带领全市人民，坚持邓小平理论和“三个代表”重要思想，以科学发展观为统领，以经济建设为中心，求真务实，开拓创新，突出重点，克难求进，全市经济社会保持良好的发展势头，顺利完成了市五届人大三次会议确定的任务。

1、经济实力跨上新台阶。

预计全市实现地区生产总值922亿元，比上年增长15%；人均地区生产总值20260元。全社会固定资产投资410亿元，增长24.2%。财政总收入117亿元，同口径增长26%。城市居民人均可支配收入11379元，农民人均纯收入5200元，分别增长15.5%和11.2%。

着力抓好“双创”和“三重”，工业第一方略强势推进。实现全部工业总产值2100亿元，规模以上工业企业超过2000家，销售收入过亿元的企业超过200家。规模以上工业实现增加值388亿元、利税101亿元，分别增长23.1%、21%。完成工业技术改造投入252亿元，增长40%。新培育省级以上高新技术企业30家、产品138个。高新技术产业产值270亿元，增长43.5%。新增中国名牌产品5个。全市建筑企业完成施工总产值420亿元，增加值92亿元，分别增长14%和18%。

以增加农民收入为中心，农村经济稳步发展。实现农业增加值86亿元，增长6%。全市粮食总产量226万吨。新增市级以上龙头企业12家，累计达47家。新增农业外资项目47个，利用外资5100万美元。“菜篮子工程”建设完成预定任务。新增无公害农产品基地70万亩，新增“三品”品牌48个。宝应县建成全国有机食品基地示范县。启动全面小康村建设，首批30个先行村基本达标。土地复垦整理力度加大，实现了耕地占补动态平衡。农业综合开发、水利工程建设、农技推广、农业机械化、气象服务等工作取得新成绩。

制定发展纲要和扶持政策，服务业持续发展。实现服务业增加值318亿元，增长14.3%。社会消费品零售总额263.5亿元，增长15.6%。接待境内外游客1100万人次，实现旅游总收入102亿元，分别增长20%和32%。全年房地产投资73亿元，商品房施工面积700万平方米。全社会货物运输量稳步增长，其中港口货物吞吐量达4596万吨。信息服务业实现销售收入35.7亿元，增长10.7%。物流、中介、会展等行业发展势头良好。金融生态环境得到改善，年末存贷款余额分别比年初增加109亿元和70亿元。

2、开发开放实现新突破。

沿江开发和园区基础设施完成投入85亿元。润扬长江公路大桥建成通车，沿江高等级公路全线基本贯通。扬州港３号泊位对外开放，４号泊位建成投产。扬州出口加工区经国务院批准设立，相关基础设施初步建成。二电厂二期工程建设进展顺利，扬州发电厂扩建工程投入运行。化工、汽车及零部件、电子信息等产业集聚初具规模。沿河开发重点基础设施建设启动，安大公路先导段开工建设。南水北调东线一期工程建成，淮河流域灾后重建应急工程投入使用。成功举办了“烟花三月”国际经贸旅游节及各类招商推介活动。全年协议注册外资24.2亿美元，增长47.4%。注册外资实际到帐老口径12.5亿美元，增长53.6%；新口径5.3亿美元。新批1000万美元以上项目106个。新增民资注册资本165亿元、民资总投入402亿元，分别增长61.9%和57%。实现外贸出口19亿美元，外经营业额1.03亿美元，分别增长40.6%和20%。

3、体制机制改革取得新进展。

国有企业改制重组不断推进，企业上市工作得到加强，宝胜科创、琼花高科股权分置改革顺利完成。着手进行市区公用事业单位改革。112家市直生产经营型事业单位改革完成人员身份置换和社保接续工作。成立市国有资产监督管理委员会。调整了市区财政体制，完善了部门预算、国库集中支付和政府采购制度。农村税费改革取得明显成效，全面免征农业税及其附加。农村土地使用权流转率达13.9%。农民专业合作经济组织已有320家。

4、城乡面貌呈现新变化。

全年市区完成城市建设投资65亿元。先后拓宽改造史可法北路、渡江南路、润扬南路等19条城区和出入口道路，整治了沿山河、新城河等4条河道。实施了邗沟河、漕河、二道河绿化风光带建设和汶河路等道路街景美化亮化改造，初步建成润扬森林公园。市区新增绿地面积134万平方米。新增、改造路灯近2万盏。翻建街巷43条。新辟公交线路10条。汤汪污水处理厂二期、开发区污水处理厂一期和城市医疗废物处理中心等工程按计划推进。村镇建设步伐加快。完成了全市镇村布局规划编制工作。80个村庄实施了环境综合整治，新建成生态村18个。农村新增改水受益人数15.6万人，改厕4万座。建成农村公路1107公里。城市管理进一步加强，基层管理责任网络逐步健全，“数字城管”启动建设。成功承办了首届城市水景观建设和水环境治理国际研讨会、全省城乡建设工作会议。城市化率达48%，比上年提高1.4个百分点。

5、社会事业取得新进步。

全年实施省级以上各类科技项目385项，其中国家级科技项目124项，争取省级以上科技项目拨款1.07亿元。我市被评为全国科技进步先进市，江都、仪征、邗江被评为全国科技进步先进县（市、区）。刘秀梵、程顺和当选为中国工程院院士。义务教育全面普及，高中阶段毛入学率达82.2%。完成农村中小学“六有”工程和教育信息化工程。实施了市三中、市特殊教育学校改扩建工程，开工建设扬州中学西区校。扬子津科教园区一期工程顺利完成。全市有16所学校开办了宏志班。城乡社区卫生服务机构建设步伐加快。重点医院建设项目顺利推进。建成市区新体育馆。成功承办了全国十运会相关赛事，扬州籍参赛运动员获金牌9.75枚，金牌贡献率列全省第3位。重视人口与计划生育工作，全市近1.3万名农村计划生育群众领取奖励扶助金。实施了市级以上文物保护单位和一批古迹的解读工程，吴道台宅第基本修复开放，“双博馆”建成开馆。承办了第四届全国“四进社区”文艺展演，举办了首届“市民日”、市第五届少儿艺术节等活动。创作了一批文艺精品。城乡有线电视入户率达69%，数字电视实现试开通。全市第二轮修志工作开始启动，市档案馆被评为国家一级档案馆。

6、关注民生收到新成效。

多渠道、多形式开发就业岗位，积极为下岗失业人员提供税费减免、职业介绍、培训补贴、小额贷款担保等扶持，全年采集就业岗位7.5万个，推荐就业3.5万人，新增城镇就业4.6万人，年末城镇登记失业率为3.8%。全年新增转移输出农村劳动力5.5万人。城镇职工养老保险扩面工作力度加大，参保人数达51万人。农村养老保险平稳运行，参保人数达31.2万人。全面提高征地补偿标准，近万名被征地农民领到了基本生活保障卡。全市城镇职工基本医疗保险参保人数达59万人。新型农村合作医疗覆盖率达88.6%。城乡6.3万人纳入低保，农村1.6万名五保对象得到妥善供养。全年“慈济医院”和“慈济窗口”为3.4万人次减免、报销医药费208万元。市区1800多户低收入和“双困”家庭的住房困难得到解决，农村三年草危房改造和扶贫建房任务全面完成。新一轮帮扶经济薄弱村和贫困户工作全面展开。残疾人事业得到加强。

7、精神文明和民主法制建设取得新成绩。

深入开展文明城市、文明村镇、文明社区和文明行业等群众性精神文明创建活动。市第一人民医院等3家单位被命名为全国文明单位，江都市丁伙镇、维扬区平山村被命名为全国文明镇和文明村。高邮市菱塘回族乡被评为全国民族团结先进单位。老龄和关心下一代工作取得新进展。和谐社区创建扎实推进，基层民主管理和村民自治得到加强，政务和村务公开不断深入。双拥工作和国防教育进一步深化，国防动员支前演练取得成功。完成市人防指挥中心主体工程建设。

认真执行市人大及其常委会决议和决定，主动接受市人大及其常委会的法律监督、工作监督。接受市政协的民主监督，积极听取各民主党派、工商联和社会各界的意见和建议。办理市人大代表建议、批评、意见311件，办理市政协提案363件。广泛开展法制宣传教育，“四五”普法目标全面完成。高度重视人民群众来信来访工作，妥善处理涉及群众利益的重点问题，疏导化解社会矛盾。强化“平安扬州”创建和弱势群体的法律援助工作，深入推进社会治安综合治理，严厉打击各种犯罪，全市社会保持稳定。健全安全生产责任制，加大安全隐患整治力度，事故发生数和死亡人数实现双下降。外事、侨务、台湾事务和民族宗教等工作取得新进展。

8、公共服务型政府建设迈出新步伐。

加快推进行政管理体制改革，清理并初步确认了362项市级实施的行政许可事项，又有98项行政许可事项进入市行政办事服务中心办理。努力畅通与人民群众联系的渠道，建立健全了政府信息公开制度，开通了中国扬州政务网和“12345”政府公开电话。对城市规划、物价等与人民群众紧密相关的重要事项实行听证、公示等制度。公文交换、网上审批等电子政务投入应用。市区为民办实事和农村“八件实事”工程完成预定目标。坚持依法行政，规范行政行为，成立了市发展软环境投诉中心。加强经济社会发展的规划引导，制定了我市“十一五”发展规划和相关专项规划。

各位代表，2005年各项工作任务的完成，标志着“十五”计划目标的实现。五年来，我们坚持加快发展不动摇，积极适应宏观经济环境的新变化，战胜“非典”和洪涝等灾害，全力做好改革、发展和稳定的各项工作，全市经济社会发展步入了新阶段。过去的五年，是扬州经济综合实力迅速增强的五年。地区生产总值五年累计3422亿元，是“九五”的1.7倍，可比价年均增长12.4%。财政总收入累计380亿元，是“九五”的2.9倍，年均增长28%。过去的五年，是改革开放取得突破性进展的五年。全市国有、集体企业和生产经营型事业单位改革取得了实质性进展，政府职能加快转变，招商引资、沿江开发、园区建设取得了重大突破，沿河开发启动实施。注册外资实际到帐五年累计29亿美元，是“九五”的8.4倍，年均增长79.8%。新增民资注册资本累计398亿元，是“九五”的7.4倍，年均增长51%。过去的五年，是着力提升城市功能和品位、城乡面貌有较大变化的五年。全社会固定资产投资五年累计1311亿元，是“九五”的2.2倍，其中城市建设投入265亿元，是“九五”的5.2倍。建成润扬长江公路大桥、宁启铁路扬州段、环城高速公路等一批重大交通工程。先后创成国家卫生城市、国家环保模范城市、国家园林城市、中国人居环境奖城市。过去的五年，是城乡人民生活水平加快提高的五年。城市居民人均可支配收入年均增长11.1%，农民人均纯收入年均增长8.5%。城市居民和农民人均住房使用面积五年累计分别增加7平方米和5.6平方米。城镇登记失业率始终控制在4.5%以内。

回顾“十五”时期改革开放和经济社会发展的实践，我们有以下几点体会：一是坚持以经济建设为中心，牢固树立和认真落实科学发展观，狠抓发展第一要务，推进经济社会快速发展。二是坚持解放思想、更新观念，用改革的思路突破体制机制上的束缚，用创新的办法破解发展中的难题。三是坚持开放开发，推进沿江沿河开发和园区建设，强化招商引资，汇聚生产要素，加快产业集聚。四是坚持基础设施建设先行，大力推进城乡建设，全面提升综合竞争力。五是坚持以人为本、关注民生，着力改善人民群众生活条件，统筹城乡、人与自然、经济与社会发展，促进发展良性互动和社会安定和谐。

各位代表，“十五”时期，我市改革开放和现代化建设取得的成就，是我们坚决贯彻落实党的路线、方针、政策的结果，是全市人民开拓进取、团结奋斗的结果，也是各方面大力支持和帮助的结果。在此，我谨代表扬州市人民政府，向付出辛勤劳动的全市人民，向关心、支持和参与扬州建设的各界人士致以崇高的敬意和衷心的感谢！

在肯定成绩的同时，我们也清醒地认识到，我市经济和社会发展还存在一些不可忽视的矛盾和问题，主要表现在：重要产业、重点企业、重大项目的支撑带动作用不够明显，经济增长方式粗放的状况尚未根本扭转，持续稳定发展的基础还不牢固；城乡居民收入水平提高不快，一些与群众利益密切相关的热点、难点问题仍未得到妥善解决；统筹发展、协调发展的任务还比较艰巨，城乡基础设施和公用设施建设、环境保护、社会稳定等方面还有许多事情要做；政府机关作风还有待改进，在服务水平、办事效率等方面还存在基层和群众不够满意的地方。这些矛盾和问题，我们将在今后的工作中努力克服并认真加以解决。

“十一五”时期的发展构想

各位代表，未来的五年，我们面临着新的形势、新的机遇、新的挑战，做好今后五年的工作责任十分重大。根据市委四届九次全会的精神，我市“十一五”时期经济社会发展的总体要求是，以邓小平理论和“三个代表”重要思想为指导，以科学发展观为统领，以全面建成小康社会为目标，以跨越发展、科学发展、和谐发展为主题，以改革开放为动力，全面实施开放开发、新型工业化、城市化、富民优先、科教兴市五大战略，建设更加富裕文明秀美的新扬州。

按照这一总体要求，我市今后五年经济社会发展的主要预期目标为：地区生产总值年均增长13%以上，工业增加值年均增长15%以上。财政总收入年均增长15%以上。全社会固定资产投资年均增长18%左右。注册外资实际到帐五年累计60亿美元，新增民资注册资本五年累计1200亿元。到2010年，全市地区生产总值达1700亿元，人均地区生产总值37000元以上。城市居民人均可支配收入16000元以上，农民人均纯收入达8000元。城市化率55%以上。城镇登记失业率控制在4.5%以内。单位地区生产总值能耗比“十五”期末降低20%左右。到“十一五”期末，全市全面建成小康社会，为2020年基本实现现代化打下坚实基础。

实现“十一五”预期目标，我们必须以科学发展观统领全局，着力调整产业结构、转变增长方式，增强自主创新能力，建设资源节约型、环境友好型社会，推动经济社会持续、快速、协调发展。为此，必须把握好以下几点：

更加注重加快发展。始终坚持以经济建设为中心，切实增强加快发展的危机意识、责任意识和创新意识，倍加珍惜机遇，倍加开拓进取，倍加努力工作，保持较快的经济增长速度，提升综合实力，实现跨越发展。

更加注重深化改革、扩大开放。加大经济体制和行政管理体制改革的力度，加大推进沿江沿河开发的力度，加大招商引资、园区建设的力度，进一步整合资源、集聚优势，不断增强发展的动力和活力。

更加注重提高城市化和城市现代化水平。进一步完善提升城市综合功能，做精、做优、做美中心城市，彰显城市个性和特色。进一步增强中心城市的辐射带动作用，促进城乡资源合理流动，形成城乡统筹发展的新格局。

更加注重关注民生。坚持发展为了人民，发展依靠人民，发展成果由人民共享，千方百计提高城乡居民收入，千方百计增加公共产品和公共服务，切实解决人民群众最关心、最直接、最现实的利益问题，不断提高人民群众的满意度，努力构建和谐社会。

2006年政府工作任务

2006年是实施“十一五”规划的开局之年，开好头、起好步至关重要。全市经济和社会发展主要预期目标是：地区生产总值增长13%以上，其中一产增长5%，二产增长15%，三产增长14%。财政收入增长15%，其中一般预算收入增长16%。全社会固定资产投资增长20%。注册外资实际到帐增长30%，新增民资注册资本增长30%。外贸出口增长20%。社会消费品零售总额增长14%。城市居民人均可支配收入增长10%，农民人均纯收入增长9%。城镇登记失业率控制在4%左右。单位地区生产总值能耗下降5%。

为完成今年的预期目标，着重做好十个方面的工作：

1、着力实施工业第一方略。

加快推进“三重”工作。强化规划导向和政策激励扶持作用，力争重要产业、重点企业和重大项目建设实现新突破。积极发展石油化工、汽车及零部件、电子信息、电工电缆等八大产业，提高重要产业的集聚度和贡献份额。加快中集通华罐式车、大连化工三期工程、大洋造船和顺大多晶硅等重大项目的建设，培育新的经济增长点。积极引进战略合作伙伴，加快资源的优化配置，培植大企业、大集团。进一步健全落实领导干部与“三重”挂钩联系制度，协调解决“三重”发展过程中的突出问题。力争全年规模以上工业增加值增长16%以上，销售收入10亿元以上的企业达20家。加快工业结构调整和优化，推动企业技术改造和技术进步，全年工业技术改造投入322亿元，增长28%，实施亿元以上重点技改项目96个。

加快实施名牌带动战略。鼓励企业增强名牌意识，积极实施争创名牌发展规划，支持企业做大做强主导产品和拳头产品，支持企业推行与国际接轨的质量、环保认证体系，以优质产品和服务不断扩大产品知名度和市场占有率，提升“扬州制造”形象。全年新创中国名牌和国家驰名商标5个，省名牌和著名商标40个。

加快建设高素质的企业家队伍。通过多渠道、多形式的教育培训，着力提升企业管理人才的素质和能力。指导各类企业建立健全激励机制，完善与现代企业制度相适应的经营管理人员的薪酬制度。培养和引进适应经济发展需要的现代化企业管理人才，推进企业家队伍职业化、市场化，营造有利于企业家健康成长的社会环境。

加快发展建筑业。积极开拓市场，提高装备水平，扩大产业规模，壮大骨干企业，提升市场竞争能力和企业形象。加大建筑业改革力度，全面完成市、县重点建筑企业的改制任务。全年建筑企业实现施工总产值、增加值均增长15%以上。

2、全面推进社会主义新农村建设。

按照统筹城乡发展的要求，制定出台《社会主义新农村建设实施意见》，着力提高农业综合生产能力，提升农村内生发展动力，加快农民致富步伐。

持续较快增加农民收入。继续推进农业结构调整，加快发展现代高效农业。稳定粮食种植面积，新增蔬菜、花木、经济林茶果20万亩。进一步实施“三品三创”工程，新建无公害农产品基地60万亩。推广新型实用技术，超高茬麦套稻扩大到40万亩以上。提升农业产业化水平，新增市级以上龙头企业3家，培植销售收入10亿元以上龙头企业2家。促进发展农产品深度加工，提高农产品附加值和市场占有率。推进乡镇工业集中区建设，引导乡镇工业集聚化发展。加大农业招商引资力度，全年利用“三资”22亿元以上。加强农村劳动力专业技能培训和劳务市场建设，全年培训农村劳动力5.5万人以上，新增劳务输出3万人以上。

积极改善农村生产生活条件。修编镇村总体规划，加强重点中心镇建设，逐步调整缩减自然村，引导农民集中居住。加大对农业和农村的投入，健全农业技术推广、农业信息服务、农产品质量安全和动植物病虫害防控体系。加快农田水利工程建设，完善农村水利设施。抓好农业综合开发。提高农业机械化水平。大力推进县乡村道路建设，改善农村道路通行条件。以创建卫生村镇为抓手，以清洁河道、清理垃圾为重点，开展农村环境综合整治。继续推进农村改水和改厕工作，全市农村自来水普及率96%以上，卫生厕所覆盖率68%以上。发挥小康先行村典型示范带动作用，推进120个全面小康村创建达标。

加强农村社会保障体系建设。完善以县为主的农村义务教育管理体制，实行农村义务教育免收杂费，继续对贫困家庭学生提供免费课本和寄宿生活费补助。健全新型农村合作医疗制度，覆盖率稳定在85%以上。完善农村最低生活保障制度，努力做到分类施保、应保尽保。实施五保户“关爱工程”，集中供养率45%以上。加快推进被征地农民基本生活保障工作，做到“即征即保”。切实做好经济薄弱村和贫困户的帮扶工作。

深化农村综合改革。稳妥推进乡镇机构改革，优化整合事业单位。加强县乡财政体制和管理方式改革，提高自我保障能力。建立健全财政支农资金的稳定增长机制。大力发展农民经济合作组织，积极扶持农村社区股份合作制，稳妥发展土地合作组织。深化农村金融体制改革，鼓励有条件的信用联社组建农村合作银行。认真搞好第二次全国农业普查工作。

3、大力发展现代服务业。

突出发展现代物流业。完成扬州港、扬州火车货运站和仪征石化等特色物流园区规划的编制，加大物流项目包装和招商引资力度。推进物流园区基础设施和信息公共平台建设。落实现代物流业发展的扶持政策，改造提升传统物流业，引进和培育一批现代物流企业。

发展壮大旅游业。科学开发、合理保护扬州古城、古运河和蜀冈—瘦西湖风景区的旅游资源，创响文化、休闲、生态特色旅游品牌。整合旅游资源，促进市与县、扬州与周边城市的旅游联动发展。加强对江浙沪地区、火车开通沿线城市及海外旅游市场的宣传促销和扬州城市的整体推介，不断提高知名度，吸引更多客源。加快旅游业与娱乐、餐饮、购物、交通等相关行业的融合。开工建设1-2家五星级饭店。全年接待境内外游客超过1200万人次，实现旅游总收入120亿元。

做优做特商贸服务业。改造提升传统商贸业，规划建设国庆路老字号一条街，打造名品名店集聚区。鼓励老字号企业发展特许经营、连锁经营、加盟经营等新型业态，放大品牌效应。通过开展“扬州师傅”评选等活动，提升服务的质量和水平，增强特色传统服务业的市场竞争力。合理优化大型连锁超市、专业市场的空间布局。加强农村商业网点建设，开拓农村市场，扩大农村消费。

拓宽服务业发展领域。加快发展信息服务业，启动扬州软件与信息服务业创业中心建设，积极申报省级软件产业基地。加强对房地产市场的引导和调控，促进房地产业健康持续发展，形成商品房、经济适用房、拆迁和解危安置房、廉租房等多层次的住房供给体系。放手发展养老托幼、体育健身、物业管理等便民利民的社区服务业。积极发展金融、保险、审计、科技服务、法律服务、会展等行业和创意产业。

4、继续推进开放开发。

大力发展开放型经济。精心组织2006“烟花三月”国际经贸旅游节，有针对性地开展各类招商引资活动，加强对千万美元以上、重点产业链、增资扩股等项目的引进，努力实现重大引资项目的新突破。拓宽招商引资领域，引导外资投向现代服务业、高新技术产业、现代农业和环保产业。建立项目推进制度，强化跟踪服务，进一步优化投资软环境，提高项目签约率、外资到帐率和开工投产率。全年新批千万美元以上项目120个。优化出口商品结构，扩大高新技术产品、机电产品、轻纺产品和农产品的出口。促进外贸企业的联合、兼并和重组，形成综合优势。鼓励有条件的企业“走出去”，全力推进对外承包合作，提高总承包在工程承包中的比重。规范境外劳务合作市场管理，扩大劳务输出规模。

加快沿江开发和园区建设步伐。全面建成沿江高等级公路，逐步完善沿江地区路网，加快港口、供电供热、污水处理等基础设施建设和生态环境建设。继续着力推进5家省级开发区和扬州化学工业园等重点园区建设，加大扬州出口加工区、市开发区光电产业园、仪征汽车工业园和邗江国家级数控金属板材加工基地等载体的招商引资力度，推动优势产业和企业向园区集聚，提高单位土地面积的投资强度，提升沿江开发、园区建设的水平。全年沿江地区工业产值、注册外资实际到帐等主要经济指标占全市总量的比重保持在80%以上。

促进区域统筹发展。推动工业化、城镇化和农业产业化互动发展，增强县域经济发展活力。出台沿河开发总体规划。继续推进安大公路和京杭运河“三改二”航道整治等工程建设，改善沿河地区基础设施条件。培植块状经济优势，打造电工电器、纺织服装、机械制造、轻工工艺等特色产业集聚区。扩大与长三角及南京都市圈城市的区域合作，加快制造业、服务业、基础设施等方面的对接，促进优势互补，资源共享。

5、深化体制机制改革。

继续推进国有集体企业改革。加快扬柴、亚星、扬农等一批企业的战略重组和增量改制，增强骨干企业的活力和竞争优势。深化产权制度改革，完善法人治理结构，做好市属企业改革扫尾工作。加快企业的股份有限公司改造，落实企业上市扶持政策，鼓励优势企业在境内外上市融资，完成联环药业、扬农化工股权分置改革。建立并完善国有资产监督管理体制，维护出资人和职工的合法权益。

发展壮大民营经济。大力推进全民创业，进一步降低投资门槛，放宽投资政策，确保各项扶持政策落实到位。引导民营企业加强制度、管理、技术和品牌创新，提升发展层次和水平，不断做大做强。支持民营企业参与国有企业改革改制，鼓励民营资本进入基础设施、公用事业、社会事业等领域。加强和改善对民营经济的服务，协调解决用地、用水、用电、融资等方面的困难，建立健全中小企业信息咨询、人才培训、技术支持等社会化服务体系，为民营经济发展创造平等竞争的环境。全年新增民资注册资本200亿元，民资投入450亿元。

深化财税、金融和投融资体制改革。调整财政支出结构，完善公共财政管理体制。稳步实施财税改革，积极培育壮大财源，大力组织财政收入。进一步规范投资项目核准权限，简化投资备案程序。推行多种形式的基础设施项目经营权和收益权的有偿转让，试行政府投资项目决策责任制度和项目代建制度。加强信用体系建设，进一步改善金融生态环境。大力支持地方法人金融机构改革，积极引进市外银行来扬设立分支机构，稳步发展融资担保主体。

继续搞好事业单位改革。在完成生产经营型事业单位转企改制扫尾工作的基础上，稳步推行以规范机构职能为重点的行政管理型事业单位改革，有序推进以完善人事、分配制度为重点的社会公益类事业单位改革。

6、不断增强科技创新能力。

逐步建立并完善科技创新体系。加强以企业为主体、市场为导向、产学研结合的创新体系建设，新培育省级以上高新技术企业40家、产品120个，组织实施省级以上各类科技计划项目350项。鼓励有条件的企业与国内外高等院校、科研院所共建技术创新联合体，搞好引进技术的消化吸收和再创新，推动各类科技资源向骨干企业集聚，新建企业技术中心、工程技术研究中心40家。加快科技成果向现实生产力转化，推进太阳能光伏和半导体照明等高科技产业基地建设，实现高新技术产业产值320亿元。发挥扬州国家级创业服务中心、留学生创业园等科技孵化器的作用，吸引一批高新技术人才入园创业。充分利用驻扬高校、科研院所和博士后工作站的优势，提高自主创新能力。

加大科技政策支持力度。认真贯彻落实全国科学技术大会精神，制定鼓励扶持政策，在全社会形成尊重科学、尊重知识、尊重人才、鼓励创新的浓厚氛围。积极争取国家和省资金支持，稳步增加各级财政对科技进步的投入，全年预算内科技三项费用增长20%左右。

加强创新人才队伍建设。制定和实施相关政策，引进和培养一批掌握核心技术、拥有发明成果的拔尖人才，引进和培养一批善于组织科技研发团队、能够统筹科技资源与生产要素配置的领军人才。突出抓好专业技术、企业管理和实用人才队伍建设，充分发挥现有人才的创新潜能。建立健全人才资源管理和开发体制，实行人力资本作价入股，加快形成鼓励各类人才提高创新能力和创新效率的收入分配机制。

7、努力提升城市建设水平。

提高城市规划水平。加快调整和完善“一体两翼”城市发展结构规划，编制扬州主城区城市总体设计、重点地段和近期建设地段控制性详细规划，完成土地利用总体规划修编，高起点、高质量推进规划全覆盖。加强与境内外高水平规划设计机构的合作，促进城乡规划设计水平的提升。强化规划集中统一管理，确保规划的权威性和严肃性。

加大古城保护力度。制定完善古城保护办法，出台老城区街坊整治等政策意见。按照老城区控制性详细规划，具体制定“三线”、“三片”保护利用详细规划。精心实施“双东”街区、教场、康山园等古城修复项目。结合保护历史建筑，推进“博物馆之城”建设，修缮重宁寺，启动天宁寺博物馆和淮扬菜博物馆建设。挖掘和利用历史、民居、宗教、工艺、休闲等文化资源，进一步展示名城魅力。

加强城市建设和综合整治。建设新扬圩路、润扬北路，延伸文昌东路、平山堂西路，整治联谊路，改造皮市街，建设三湾大桥、文昌大桥，新建城东、城北公交客运中心。完善古运河沿线等绿化风光带，新增城市绿地100万平方米以上。分片区改造老城区综合管网，翻建街巷40条。拆除市燃气总公司焦化厂，建设天然气门站。继续抓好新城西区基础设施建设；稳步推进蜀冈—瘦西湖风景区基础设施和项目建设；高起点编制城市东区规划，启动重点项目建设。

提升城市管理水平。强化区级城管职能，充实加强城市基层管理力量，落实街道、社区管理责任制，实现城市管理属地化。启动运行数字化城管新体制，完善城管行政执法和监督体系，深入开展城管创优活动，综合运用行政、法律、科技、市场等手段，提高城管处置能力和长效管理水平。

搞好生态环境保护。大力发展循环经济和生态工业园，万元GDP污染物排放量下降10%。督促指导30家重点企业实施清洁生产并通过审核，创建1—2家省级以上环境友好企业、3个省级绿色社区。进一步加强南水北调东线源头和集中式饮用水源地水质保护，防范重点区域、水域环境风险。强化环境执法监督，实施排放总量控制、排放许可和环境影响评价制度。实施最严格的耕地保护制度，切实提高土地集约利用水平。加大土地复垦开发和整理力度，保持全市耕地占补动态平衡。全年植树造林10万亩，森林覆盖率提高1个百分点。

8、协调发展各项社会事业。

坚持教育优先发展。努力办好人民满意的基础教育。高水平、高质量普及九年义务教育，加快普及高中阶段教育和学前教育。全面实施素质教育，积极推进新课程改革，不断提高教学质量。优化教育布局，提升办学质态，放大优质教育资源，提高教育均衡发展水平。进一步落实“两免一补”政策，扩大宏志班办学规模。加大财政对义务教育的投入，严格规范教育收费行为，鼓励社会投资办学。大力发展以就业创业为导向的职业教育。加快培养高素质技能型人才，努力打造全国特色服务业和长三角制造业的职业技术教育基地。抓好扬子津科教园二期、市职业高中新校区建设。积极支持扬州大学和地方高校建设。

进一步完善公共卫生体制和卫生设施条件。建立并完善疾病预防控制、医疗救治、卫生监督和公共卫生信息网络体系。改善卫生基础设施条件，加快建设市急救中心综合楼、扬州南方协和医院主楼、苏北医院病房楼、妇幼保健院医技楼等一批医疗设施项目，建设市传染病医院，启动中医院改造工程。基本建成城乡社区卫生服务体系。做好重点传染病防治和城乡爱国卫生工作。围绕人民群众关注的看病难、看病贵问题，整顿和规范医疗服务秩序，加强医德医风建设和药品价格管理，提高医疗服务质量。

积极发展文化体育等事业。加强市、县公共图书馆和基层文化设施建设。办好省第四届扬剧节、市第三届农民艺术节、“市民日”等活动，创作一批群众喜闻乐见的文艺精品。加强文化市场管理、文物保护和民俗民间文化保护工作。办好国际竞走挑战赛、全国举重锦标赛和市第五届全民健身节。建设体育公园国家级全民健身活动中心。发展广播电视事业和产业，城乡有线电视入户率达73%。加强人口与计划生育工作，进一步落实计划生育奖励扶助政策，人口出生率控制在7‰以内。继续做好社科研究、档案、方志、气象和防震等工作。

9、进一步提高人民群众的生活水平和质量。

积极扩大就业和再就业。实施积极的就业政策，千方百计增加就业岗位，鼓励企业吸纳就业、创业带动就业、职工自谋职业，全年采集就业岗位8万个，新增城镇就业2.7万人，帮助下岗失业人员实现再就业1.5万人。健全再就业援助机制，重点帮助“4050”人员和特困家庭新增劳动力实现就业再就业。重视做好失地无业农民的再就业工作。合理引导和规范企业裁员，严格控制失业率。建设覆盖城乡的就业管理服务体系，统筹管理城乡劳动力资源和就业工作。充分发挥劳动密集型企业、中小企业和社区服务业在促进就业再就业方面的作用。加强职业技能培训，全年培训3万人。

健全完善多层次的社会保障体系。继续推进社会保险对城镇各类企业职工、个体工商户和灵活就业人员的全覆盖，全年养老保险净增缴费人数4万人以上。调整企业职工养老金个人帐户规模。实行市区社会保险统筹。扩大医疗保险覆盖面。继续做好城市低保工作。加快推进市职介中心、社保中心、信息中心建设项目和“金保工程”建设。建立城镇最低收入家庭廉租房保障制度，继续搞好危房改造。加大医疗救助力度，进一步办好“慈济医院”和“慈济窗口”。健全教育帮扶制度，建立扶贫助学长效机制。重视退伍士兵安置和优抚对象的抚恤、优待工作。加强社会保障公共服务体系建设，多渠道筹措社会保障基金，提高社会保障能力。积极发展社会慈善事业，继续开展社会捐赠、互助活动。加强法律援助，依法维护弱势群体的合法权益。重视做好助残工作。

努力改善城乡居民生活。建立健全收入增长机制，使职工工资随着企业效益提高而提高。普遍推行工资集体协商，严格执行最低工资标准，逐步建立企业欠薪保障制度。强化政府对收入分配的调节，完善利益分配和协调机制，千方百计增加城乡居民特别是中低收入者的收入，切实维护广大职工和外来务工人员的合法权益，促进人民群众安居乐业。

维护人民生命财产安全。全面落实安全生产责任制，建立和完善防范重特大安全生产事故的责任与追究机制，加大重大安全隐患的治理力度。抓好安全标准化示范工作，增加公共安全设施投入。制定全市突发公共事件应急预案，提高保障公共安全、处置突发事件和抗击灾害事故的能力。切实加强食品、药品及公共场所卫生监督管理工作。

10、加强精神文明和民主法制建设。

抓好精神文明建设。广泛深入开展文明城市、文明村镇、文明社区和文明行业创建活动。大力推进公民道德建设，继续实施现代市民和现代农民两大教育工程，提升社会公德、职业道德和家庭美德水平。大力培育和弘扬创业、创新、创优精神，为经济社会发展提供强大的精神动力。深化爱国主义、集体主义、社会主义教育。完善未成年人思想道德建设长效机制，关心下一代健康成长。重视做好老龄工作。推进全社会诚信建设。加强双拥和国防动员工作，争创省和国家“双拥模范城”。

推进民主法制建设。认真落实市人大及其常委会的各项决议、决定，及时报告工作。支持人民政协更好地履行政治协商、民主监督和参政议政职能。提高人大代表议案、建议和政协提案的办理质量。重视社会各界提出的要求和批评建议。开展“五五”普法教育和“法治扬州”建设，积极营造良好的法制环境。进一步贯彻国务院《全面推进依法行政实施纲要》，落实行政执法责任制，强化层级监督和专项监督，不断提高依法行政水平。加强基层民主建设，健全基层民主自治制度，推进村务和社区事务公开。重视做好民族宗教工作。

切实维护社会稳定。深入开展“平安扬州”创建活动，严密社会治安防控体系，严厉打击各种犯罪，全面落实综合治理各项措施，维护良好的社会治安环境。切实做好人民来信来访工作，加强社会稳定预警以及矛盾纠纷的排查调处、应急处置、责任追究等机制建设，预防和妥善处置各类群体性事件，努力化解人民内部矛盾。

推进公共服务型政府建设

“十一五”时期，扬州实现跨越发展、科学发展、和谐发展，要求各级政府切实解放思想，转变职能，创新工作，不断提高公共管理和公共服务能力，为经济社会的持续健康协调发展提供重要保证。

1、进一步转变政府职能。

深入贯彻《行政许可法》，按照政企分开、政资分开、政事分开和政府与中介组织分开的原则，加快转变政府职能。继续推进行政审批制度改革，依法减少和规范行政审批，加强行政办事服务中心建设，做到行政审批职能集中到窗口，授权到窗口，办结在窗口。增加财政对公共领域的投入，强化政府提供公共产品和服务的职能，提高财政性资金的使用效率。依法加强市场监管，切实维护好公平竞争的市场秩序。协调整合社会资源，积极构建各类产业项目落户平台、银企信息交流沟通平台、人力资源服务平台等，推进各类资源加速向扬州集聚。进一步规范发展社会中介组织，积极发挥行业协会、商会的作用。

2、切实改进作风。

大兴求真务实之风，把加强调查研究作为提升能力的重要环节，深入实际研究全市经济社会发展中的重大问题，提高政府工作的效率。推进行政管理决策的科学化、民主化，健全对涉及经济社会发展全局重大事项决策的协商和协调机制，健全对专业性、技术性较强重大事项决策的专家论证、技术咨询、决策评估制度，健全对与人民群众利益密切相关重大事项决策的公示、听证制度。狠抓各项工作的落实，围绕全面建成小康社会的目标任务，形成一级抓一级、层层抓落实的工作机制，提高政府机关的执行力。加强行政效能建设，办好发展软环境投诉中心和政府公开电话、“寄语市长”等，受理和解决好市民与投资者对经济社会发展等方面的诉求。加强便民服务中心建设，年内市区所有街道都建立便民服务中心。

3、加强公务员队伍建设。

教育引导广大公务员不断解放思想，开阔眼界，开阔胸襟，开阔思路，创造性地开展工作、搞好服务。结合《公务员法》实施，深入开展学习型机关、学习型干部创建活动，引导广大公务员不断学习，创新思维，增长才干。以全面落实领导干部廉洁自律各项规定为重点，健全完善惩治和预防腐败的各项制度规定，加强行政监察、财政监督和审计监督，堵塞可能发生腐败的漏洞。加强行风建设，推进从严治政，加大违纪违法案件查处力度。加强对土地征用、房屋拆迁、企业改制重组和破产中侵害群众利益行为以及教育乱收费、医药购销、食品安全等问题的专项整治，坚决纠正损害群众利益的不正之风。牢记“两个务必”，带头厉行节约，制止铺张浪费，以节约型政府建设推动节约型社会建设。牢固树立执政为民的理念，坚持把人民群众关心的热点、难点问题作为政府工作的重点，把群众需求作为政府工作的第一信号，把亲民、为民、惠民、富民作为政府工作的基本准则，努力实现好、维护好、发展好人民群众的根本利益。

各位代表，我们已经迈上“十一五”发展的新征程。让我们在中共扬州市委的领导下，高举邓小平理论和“三个代表”重要思想伟大旗帜，以科学发展观统领经济社会发展全局，团结和带领全市人民，开拓创新，锐意进取，真抓实干，奋发有为，为加快实现全面建成小康社会的宏伟目标而努力奋斗！
